# Identifying avoidable causes of perinatal deaths in a district hospital in Lesotho

**DOI:** 10.4102/curationis.v47i1.2497

**Published:** 2024-02-29

**Authors:** Rose Nonyane, Emmerentia du Plessis, Jeannette Clase

**Affiliations:** 1 NuMIQ / School of Nursing Science, Faculty of Health Sciences, North-West University, Potchefstroom, South Africa

**Keywords:** female, hospitals, district, perinatal deaths, pregnancy, preventable, retrospective studies

## Abstract

**Background:**

Certain determinants can be associated with avoidable perinatal deaths, and audits are needed to establish what these determinants are, and what can be done to prevent such deaths.

**Objectives:**

The study aimed at identifying and describing determinants associated with avoidable perinatal deaths at a district hospital in Lesotho and strategies to curb their occurrence.

**Method:**

A retrospective descriptive study was conducted using 142 anonymised obstetric records from January 2018 to December 2020. A data collection tool was adopted from the Perinatal Problem Identification Programme. In this tool, avoidable determinants are referred to as ‘factors’ or ‘problems’.

**Results:**

A concerning number of perinatal deaths were secondary to avoidable patient factors, namely a delay in seeking medical care, inappropriate responses to antepartum haemorrhage, and inadequate responses to poor foetal movements. Medical personnel factors are also worth observing, namely incorrect use of partograph, insufficient notes to comment on avoidable factors and ‘other’ medical personnel problems. Ranking highest among administrative problems were the unavailability of intensive care unit beds and ventilators and inadequate resuscitation equipment. Administrative problems accounted for more perinatal deaths than the patient-related factors and medical personnel factors.

**Conclusion:**

There is an urgent need for periodic audits, health education for patients, staff competency and the necessary equipment to resuscitate neonates.

**Contribution:**

Avoidable determinants associated with perinatal deaths in a district hospital in Lesotho could be identified. This information provides an understanding of what can be done to limit avoidable perinatal deaths.

## Introduction

In the prenatal period, namely before birth, during pregnancy, and shortly after birth, reducing neonatal mortality is crucial in attaining the third Sustainable Development Goal (SDG), that countries should aim to reduce neonatal mortality to at least as low as 12 per 1000 live births by 2030 (United Nations Children’s Fund [UNICEF] 2019). However, globally neonatal mortality is still of concern (Ashish et al. 2016; Nijkamp et al. 2017; World Health Organization [WHO] 2020). Around 77% of global stillbirths occurred in sub-Saharan Africa and South Asia in 2019, yet most of them are preventable through access to quality care before or during labour (Suzuki & Kashiwase 2020). Regardless of the high number of perinatal deaths, namely a stillbirth or a death occurring in the first week of life (Fraser, Nolte & Myles 2015), adequate action with respect to this issue is still deficient (Blencowe et al. 2016).

The high number of perinatal deaths globally is a call for active perinatal audits to ascertain causes associated with individual deaths; additionally, there is a need to establish which prevention interventions work (Aminu, Bar-Zeev & Van der Broek 2017; Po et al. 2019). Perinatal audits are highly important for preventing high rates of avoidable perinatal deaths, namely all deaths avoidable through adequate care, which are associated with patient and medical personnel problems. Mdoe et al. (2022) have observed that to reduce these high rates of death, patients should be advised of the importance of attending antenatal care and facilities should improve maternal and neonatal care.

Many perinatal deaths are avoidable. In a study conducted in Brazil, a total of 2210 foetal deaths occurred over a 10-year period, 80% of which were preventable (Bonfirm et al. 2020). This can be seen as a call for evidence-based interventions that will reduce the occurrence. Kashilika and Moshi (2021) assert that when effectively used, a Maternal and Perinatal Death Surveillance and Response (MPDSR) system is the answer in the fight against perinatal deaths from avoidable causes – hence the need to adopt it. Kinney et al. (2020) have also emphasised the importance of MPDSR because it is a system aimed at understanding and addressing maternal and perinatal deaths to prevent future deaths. There was thus a need to identify causes associated with avoidable deaths; there is also the need to identify measures to reduce their occurrence. Identifying avoidable causes associated with perinatal deaths in a district hospital, a setting with limited resources, will be beneficial in improving foetal outcomes.

The WHO (2022) *New-born Mortality* Report finds that most new-born deaths take place in low- and middle-income countries, such as Lesotho. Of specific concern is that in 2015 in Lesotho, when 60 000 babies were born (equating to 200 births daily), three stillbirths (1.5%) occurred daily, and each day six babies (3%) under 1 month died (UNICEF 2015). Clinical management is of great importance after a perinatal death; it requires appropriate investigation and assessment to determine causes and thereby prevent future losses (Gardiner et al. 2016). Perinatal mortality is an important outcome indicator for a country’s socioeconomic situation and quality of life. These determinants directly mirror the quality of prenatal, intrapartum and newborn care – hence the need to conduct the study in Lesotho (Madaj et al. 2017).

Research was thus needed on causes associated with avoidable deaths in Lesotho (as documented in the anonymised copies of obstetric records) to understand and address causes associated with perinatal deaths to prevent future deaths. The researchers argue that if these causes are identified and described at a specific district hospital in Lesotho, recommendations can be formulated on how to reduce avoidable perinatal deaths, including stillbirths and early neonatal deaths.

## Research question

What are the determinants associated with avoidable deaths as documented in the anonymised copies of obstetric records in a district hospital in Lesotho?

## Conceptual definitions

**Perinatal deaths** – The death of a baby between 28 weeks of gestation and the first week of life (Dessu & Dawit 2020).

**Avoidable perinatal deaths** – ‘all deaths avoidable by adequate care, quality assistance, more specifically in terms of early diagnosis and effective interventions’ (Regoa et al. 2018).

**Stillbirth** – ‘a death occurring after 22 completed weeks of gestation or more, or weighing at least 500 grams’ (Musafili et al. 2017).

**Neonatal death** – ‘death among live births during the first 28 completed days of life (noting that there can be early or late neonatal deaths)’ (Fraser et al. 2015).

**District hospital** – a hospital that admits referrals from clinics and community health centres and provides general healthcare (KwaZulu-Natal Province Health 2023). A district hospital may have between 50 and 600 beds and must serve a defined population within a health district and support primary healthcare (*National Health Act* 2003). In this regard it was a district hospital in Lesotho (name withheld).

## Objective of the study

To identify and describe determinants associated with avoidable perinatal deaths in a district hospital in Lesotho and to formulate recommendations to reduce avoidable perinatal deaths.

## Methodology

### Research design

The study employed a quantitative retrospective descriptive design (Ranganathan & Aggarwal 2018). This design is defined as a quantitative method that uses data that have already been collected, typically through clinical charts, about events that have already occurred (Houser 2023). The design suited this study, as the outcome of interest, in this case recorded perinatal deaths, has already occurred (Ranganathan & Aggarwal 2018). An all-inclusive sample was used, namely all obstetric recors with a documented perinatal death for the period from January 2018 to December 2021. Retrospective data from anonymised obstetric records was used to extract data on determinants associated with avoidable perinatal deaths at a district hospital in Lesotho.

### Study setting

The study setting was a district hospital. The district hospital is situated in one of the 10 health districts in Lesotho, and this district ranks as having one of the highest perinatal death rates in Lesotho. The hospital serves as the referral hospital for the 19 clinics in the district. The hospital has one maternity unit and a nursery, and, at the time of the study, had one obstetrician, one advanced midwife, and five nurse midwives working in the maternity unit. The hospital serves a population of 61 000 people and is a 148-bed hospital (UNICEF 2017). The gatekeeper, namely the District Hospital Management Officer, granted the researcher permission to conduct the research. This process is described in more detail under ‘Data collection’ and ‘Ethical considerations’.

### Population and sampling

The population comprised all the obstetric records of a documented perinatal death for the period starting from January 2018 to December 2020. The study used an all-inclusive sampling of all records with documented stillbirths and perinatal deaths during the above-mentioned period. No participants were engaged, and the 142 records met the eligibility criteria of the study. The eligibility criteria entailed that only the following obstetric records were included: obstetric records of women who delivered in a district hospital in Lesotho from 01 January 2018 to December 2020 with a perinatal death documented in the obstetric record. The obstetric records of perinatal deaths that occurred 7 days or longer after birth were not included as the study focused only on the period when most deaths occur. Confidentiality of information and the process of obtaining permission to conduct the study and accessing the records are explained under ‘Data collection’ and ‘Ethical considerations’.

### Data collection

Permission to conduct the study was granted by the Ministry of Health of Lesotho and the relevant District Hospital Management Officer. Furthermore, goodwill permission to conduct the study was sought from the nursing manager of the maternity unit. The researcher then obtained the assistance of the hospital’s data information officer. The researcher and the information officer screened all records to identify eligible records according to eligibility criteria. Data were collected over 10 days at the hospital, using anonymised records.

Data were collected by the researcher, capturing it into a spreadsheet adopted from the standardised data capturing form of the Perinatal Problem Identification Programme (PPIP). The PPIP was developed as an audit tool for perinatal deaths and to establish a national database of the causes of perinatal deaths, to inform quality improvement initiatives (Rhoda et al. 2014). In the standardised data capturing form of the PPIP, determinants of avoidable perinatal deaths are listed as ‘avoidable factors/problems’, namely patient associated factors, administrative problems, and medical personnel associated factors, with several items listed under each of these groupings. There is also a category ‘insufficient notes to comment on avoidable factors’.

The information in the records were often not detailed, as in line with the PPIP, a coding system was used to record the causes of perinatal deaths. The information available was thus a code, with a short explanation, for example ‘inappropriate response to poor foetal movements’, without specific detail. The assigned determinants associated with a perinatal death were co-checked by the information officer of the hospital to ensure rigour. A number was assigned to every record for ease of access in case of the need for a re-check.

### Data analysis

Data were analysed by a statistician of the North-West University using IBM SPSS version 24. Descriptive data analysis was performed to formulate frequencies and enable cross tabulations. Frequencies were calculated by counting the number of stillbirths and perinatal deaths that occurred, using nominal data. The determinants associated with the preventable perinatal deaths were grouped and described in terms of frequencies and percentages. Cross-tabulations were calculated to analyse the relationship between multiple variables. This included correlations between demographic information and preventable determinants, as well as correlations between the different preventable determinants.

### Validity and reliability

The PPIP is a scientifically approved quality improvement and monitoring tool used to assess preventable perinatal deaths to curb their occurrence (Rhoda et al. 2014). The tool used in this research has been used in other research studies (Malinga 2017). Through double-checking by the research team and relying on the built-in computerised validation checks that improve the quality of data (Rhoda et al. 2014), the tool was deemed valid and reliable. As there are no variables in the tool that were measured through a Likert scale, no statistical validity and reliability coefficients could be determined for this tool (Malinga 2017). To ensure the validity of the study results, the researcher worked with the hospital information officer to double-check the data and assign a cause of death, thus ensuring accuracy. In addition, on the first day of data collection a trial run was conducted, namely that the researcher collected data from five copies of anonymised obstetric records and sent it to the statistician, the supervisor, and co-supervisor who verified that the data were captured correctly and that the tool was effective to be used for the data collection. The spreadsheet was assessed by the supervisor, the co-supervisor, and the statistician (all research experts) to ensure data reliability.

### Ethical considerations

A scientific committee, the Health Research Ethics Committee of the North-West University (Referral number NWU-00492- 20-A1) and Lesotho Ethics Board approved the study; furthermore, goodwill permission was obtained from the district hospital. Obstetric records were anonymised by removing identifiers to protect patients’ privacy. A confidentiality agreement was signed between the researcher, statistician, supervisor, co-supervisor and information officer. Data were captured on the researcher’s password-protected personal laptop and external hard drive for backup to ensure confidentiality and will be deleted after 5 years as per university rules.

## Results

**FIGURE 1:**
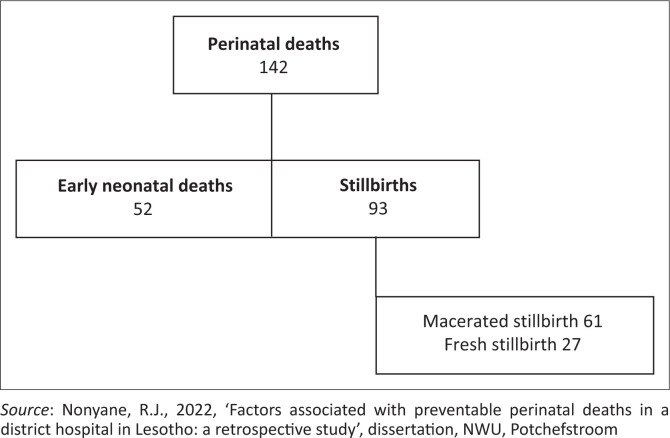
Outline of perinatal deaths.

**FIGURE 2:**
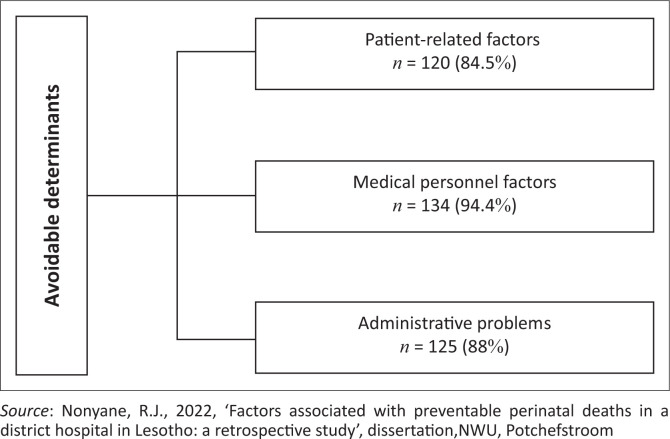
Determinants associated with preventable perinatal deaths.

**FIGURE 3:**
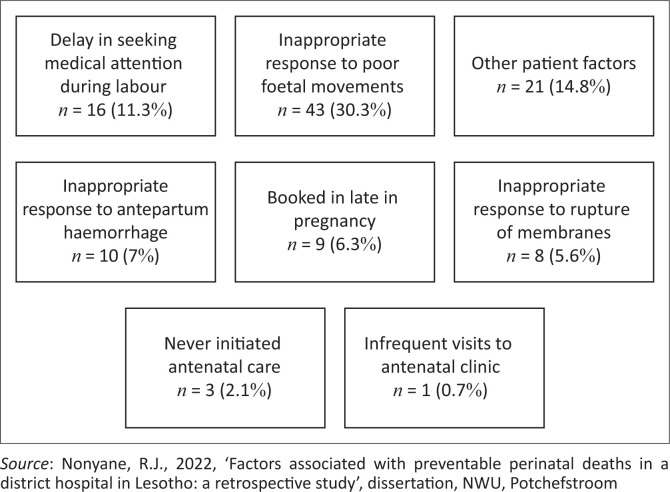
Patient-related factors associated with perinatal deaths.

The results are discussed next using figures and tables to present the findings; the latter comprise actual cases of perinatal deaths that occurred in the district hospital, demographic data of mothers and neonates who experienced a perinatal death and rates of avoidable determinants associated with the perinatal deaths. Firstly, an overall outline of the recorded perinatal deaths is presented (Figure 1). Secondly, this is followed by the demographic data of mothers and neonates as recorded (Table 1). Thereafter, the results per broad category of avoidable determinants are outlined, titled patient-related, medical personnel and administrative problems (Figure 2) as in the PPIP. Lastly, a detailed outline of the results per broad category of avoidable determinants (Figure 3 to Figure 5) is provided.

**FIGURE 4:**
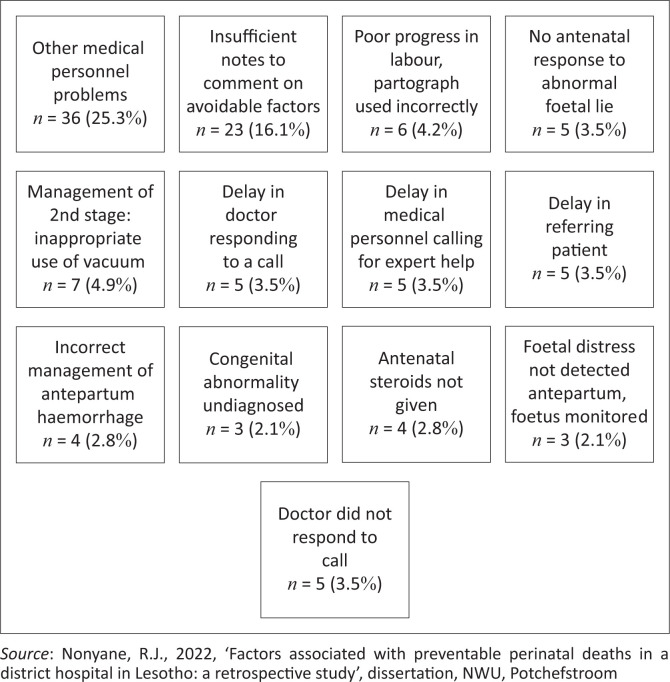
Medical personnel factors associated with perinatal deaths.

**FIGURE 5:**
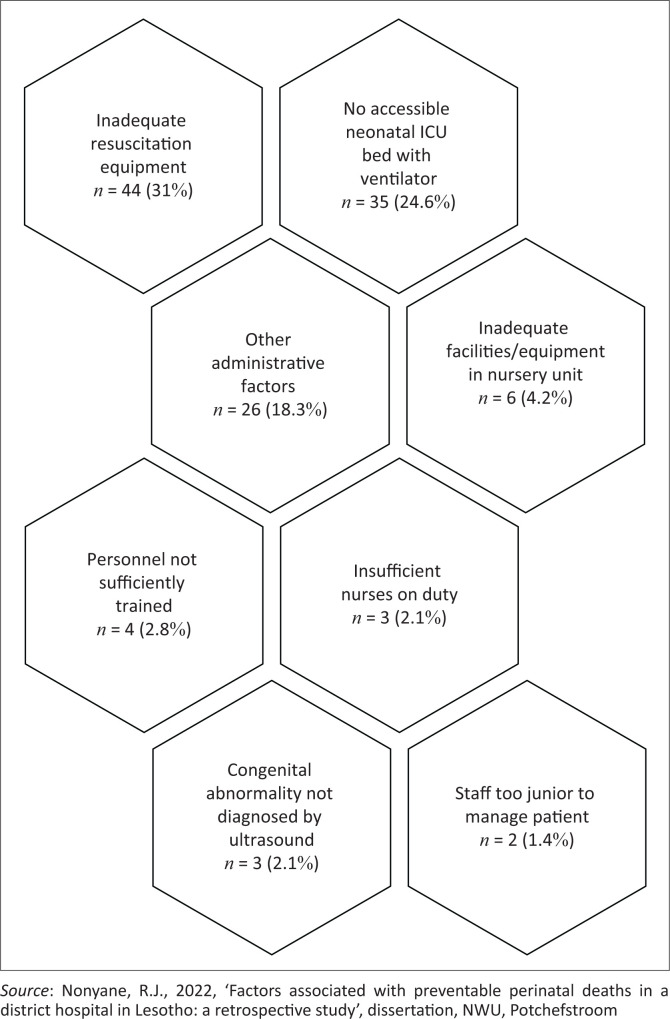
Administrative problems associated with perinatal deaths.

**TABLE 1:** Demographic data of mothers and neonates (*n* = 142).

Demographic variables	Categories	Frequency	Missing (not documented)		%
			*n*	%	
Birthplace	In transit	9	4	2.82	6.34
	At home	4	-	-	2.82
	At hospital	122	-	-	85.92
	At another facility	3	-	-	2.10
Birth mass	1500–1999 g	45	-	-	31.70
	2000–2400 g	34	14	9.86	23.94
	2500 g +	49	-	-	34.50
Maternal age	14–19 years	19	-	-	13.40
	20–35 years	75	12	8.45	52.80
	36 years	36	-	-	25.35
Parity	First birth	50	-	-	35.20
	Second birth	38	-	-	26.77
	Third birth	21	19	13.40	14.78
	Fourth birth	13	-	-	9.15
	Fifth birth	1	-	-	0.70
Antenatal care attendance	Attended	117	-	-	82.40
	Not attended	7	18	12.68	4.92
Condition at birth	Born alive	52	-	-	36.60
	Stillborn, alive in utero on admission	22	-	-	15.50
	Fresh stillborn, dead on admission	5	2	1.40	3.50
	Macerated stillborn	61	-	-	43.00
Pregnancy status	Singleton	133	-	-	93.70
	Multiple births	3	6	4.20	2.10
Gestation	Trimester 1	12	-	-	8.50
	Trimester 2	98	23	16.20	69.00
	Trimester 3	9	-	-	6.30

*Source:* Nonyane, R.J., 2022, ‘Factors associated with preventable perinatal deaths in a district hospital in Lesotho: a retrospective study’, dissertation, NWU, Potchefstroom

### Broad outline of recorded perinatal deaths

Figure 1 shows the broad outline of the results in terms of how many perinatal deaths were recorded, illustrating the number of early neonatal deaths versus stillbirths.

As shown in Figure 1, most of the perinatal deaths recorded were thus stillbirths.

### Demographic data of mothers and neonates

A demographic profile of mothers and neonates is presented in Table 1. The demographic parameters include age of the mother, parity, place of birth, birth weight, condition at birth, antenatal attendance, pregnancy status and stage of gestation.

Table 1 shows that most perinatal deaths occurred among women who attended antenatal clinics, who delivered in hospital, those who were delivering for the first time, with a single pregnancy, mostly for women aged 20–35 years, of new-borns with birth weights above 2500 g and in the third trimester. This is despite these circumstances theoretically having conferred a relatively improved survival rate for the foetus or infant compared with other cohorts.

### Avoidable determinants

Avoidable determinants refer to incidents associated with actions of the mother (patient-related factors), actions of the healthcare worker (medical personnel factors) and of the health system (administrative problems), which may have altered the outcome of the specific case had it been managed differently (Rhoda et al. 2014). The results regarding the avoidable determinants are presented next, firstly, per broad category, and secondly, in detail per category.

#### Avoidable determinants per category

Figure 2 outlines the overall results regarding the avoidable determinants per category.

From the results, it was determined that in most cases at least two or three of these avoidable determinants could be attributed to a perinatal death. The need to assess all categories on how they could have an association with the occurrence of perinatal death is thus emphasised and more than one category could be recorded per death.

#### Avoidable determinants per category

Figure 3 to Figure 5 indicate in detail the results per category of avoidable determinants, starting with the patient-related factors.

**Patient-related factors associated with perinatal deaths:** Figure 3 presents the patient-related factors associated with perinatal deaths, as recorded in the anonymised obstetric records.

In all the patient-related factors, ‘inappropriate response’ refers to a delay in seeking medical help or to applying ineffective remedies. Patient-related factors of concern that accounted for most perinatal deaths included an inappropriate response to poor foetal movements and delay in seeking medical attention during labour and other patient factors. ‘Other patient factors’ were not specified, but the value of noting this category is that it confirms that patient-related factors contribute significantly to perinatal deaths, and that antenatal care and health education to mothers are essential.

**Medical personnel factors associated with perinatal deaths:** Results regarding medical personnel factors associated with perinatal deaths are outlined in Figure 4.

It is of concern that medical personnel problems associated with perinatal deaths such as failure to identify poor progress in labour where the partograph was incorrectly used as well as doctors’ delay in seeking expert help, and doctors not responding to the call were observed, as these problems are high risks and avoidable. The problem of insufficient notes is also of concern, as inadequate recording derails effective auditing and consequent quality improvement initiatives (Rhoda et al. 2014). In addition, ‘other’ medical personnel problems were not specified, indicating a gap in recording, which limits effective auditing and future quality improvement initiatives (Nieuwoudt, Mackay & Mda 2022).

**Administrative problems associated with perinatal deaths:** Figure 5 contains the results regarding administrative problems associated with perinatal deaths.

Of great significance is the inadequate resuscitation equipment, no accessible intensive care unit (ICU) bed with ventilator, and inadequate equipment in nursery unit as they are associated with multiple perinatal deaths. Once again, the limitation of ‘other’ administrative problems is observed, confirming the gap in recording and consequent limited auditing (Nieuwoudt et al. 2022).

## Discussion

The study aimed to identify and describe determinants associated with avoidable perinatal deaths as documented in the anonymised copies of obstetric records in a district hospital in Lesotho. Mainly two outcomes emerged, namely the identification of avoidable determinants of perinatal deaths, and the importance of an audit for effective healthcare and resource planning.

Regarding the avoidable determinants, it was clear that a combination of patient-, medical personnel-, and administrative-related factors contributed to perinatal deaths. Regarding the patient-related determinants, the results show that a significant number of perinatal deaths occurred because of patient-related factors. More stillbirths than early neonatal deaths were recorded, with macerated stillbirths accounting for most deaths. Additionally, most women who had perinatal deaths did attend antenatal care and it mostly affected primigravidas. Sighn et al. (2020) confirm these findings as they state that stillbirths account for over half of perinatal deaths, which occur during delivery and are largely avoidable. In a study conducted in Brazil by Teixeira et al. (2019) 66.3% of early neonatal deaths could have been avoided through adequate care of women during pregnancy, labour and care of newborns. In a study conducted in Australia, stillbirths accounted for 75% of perinatal deaths with the remainder being neonatal deaths (Australia Institute of Health and Welfare 2019).

The study’s findings also reveal that patients’ delay in seeking medical help when there are limited foetal movements, antepartum haemorrhage and rupture of membranes, and delay in seeking help when the baby is sick, are all further determinants commonly associated with perinatal deaths. Mdoe et al. (2022) confirm these findings and assert that perinatal audits can lead to the identification of existing potential delays, which include decisions to seek medical care, reach the appropriate facility in time and receive timely quality care.

Of notable concern is that the most avoidable determinants of perinatal deaths were associated with medical personnel factors and administrative factors, as was also found by Taghizadeh et al. (2017). Taghizadeh et al. (2017) reported that obstetricians, gynaecologists and midwives are specialists who commonly have medical malpractice suits against them where it is claimed that the deaths are often avoidable or preventable. In line with these findings, Sighn et al. (2020) indicated that intrapartum deaths are closely linked to the quality of care. Miyoshi et al. (2019) in a study conducted in Zambia agreed that half of all perinatal deaths are associated with acute intrapartum events that could have been prevented. They further encouraged effective interventions, assessment of maternal risk and timely referral in reducing perinatal deaths.

From these findings, it is clear that strategies to curb the occurrence of avoidable perinatal deaths should address patient-related, medical personnel and administrative related factors. Vallely et al. (2021) also recommend that perinatal deaths can be avoided through a package of interventions at both the facility and community levels. This package of interventions can include, for example, placing competent staff in maternity wards and educating pregnant women on danger signs and when to seek medical help. This study shows that in the context of a district hospital in Lesotho, the strategies should include health education to primigravidas regarding monitoring foetal movements and timely seeking medical help, equipping medical personnel to identify and respond to risks in time and ensuring that there is enough appropriate equipment available to manage emergencies.

The second major outcome of the study is that the study confirmed the importance of audits to identify avoidable determinants of prenatal deaths and to plan healthcare for pregnant women, mothers and neonates in a highly effective and targeted way, especially in the context of a district hospital with limited resources. In addition, effective audits should be made possible through requiring thorough, consistent and detailed recording. In this regard, perinatal committees can play a major role in ensuring quality of recording and to implement audits for the purpose of effective healthcare and resource planning (Vallely et al. 2021).

## Strengths and limitations of the study

The strength of the study is that avoidable determinants associated with perinatal deaths in a district hospital in Lesotho could be identified, using a valid and reliable instrument and a rigorous research process, including the involvement of independent persons (the hospital information officer and university statistician). This information provides an understanding of what can be done to limit avoidable perinatal deaths. A limitation was that the data were collected from obstetric records and the authors had to rely on what was recorded, taking into consideration that recording errors may have occurred. Some of the records could not be located, and if all records had been accessed, it would have increased rigour.

## Recommendations

The research study contributes to giving the women and their unborn babies a voice in the form of recommendations that resulted from this research. Recommendations for practice include the following: Regarding the patient-related factors, it is recommended that health education to pregnant women should be given regarding danger signs and when to seek help, and they should be strongly encouraged to attend antenatal care. Regarding factors relating to medical personnel, it is imperative that medical personnel placed in maternity wards should be competent, specifically regarding the correct use of the partograph to identify poor progress in labour and when to seek expert help. Doctors should be supported and held accountable to timely responding to calls. In terms of administrative factors, all necessary equipment that save lives should be procured by all institutions conducting deliveries. A thorough, detailed and consistent recording should be required, so that effective auditing is possible, leading to effective and targeted healthcare and resource planning.

Recommendations for education is that recording, auditing, healthcare and resource planning, maternity care to identify and respond to danger signs, and multidisciplinary teamwork in identifying and responding to danger signs should be emphasised in both formal and in-service training. Finally, there is a need for further research to investigate the underlying circumstances that influence avoidable determinants, particularly qualitative research to obtain in-depth and contextual information that can add to the understanding of avoidable factors leading to perinatal deaths.

## Conclusion

Determinants associated with avoidable perinatal deaths were classified into patient-related, medical and personnel factors and administrative problems, based on the wording used in the PPIP. Patient-related factors consisted of delay in responding to poor foetal movements, delay in responding to antepartum haemorrhage and delay in seeking medical help when the baby is sick. Medical personnel problems included poor record-keeping, resulting in the inability to classify the avoidable determinants, doctors’ delay to seek expert help, delay in responding to a call or not answering a call at all. Administrative problems entailed the unavailability of ICU beds with a ventilator, inadequate resuscitation equipment and inadequate equipment in the nursery.

The research study not only contributes to an understanding of the preventable determinants associated with perinatal deaths in the relevant context but also has the potential to instil change by enabling tailor-made recommendations, thus reducing the occurrence of the most prevalent and concerning determinants. The outcomes of the research thus have the potential to transform the healthcare provided to pregnant women to prevent perinatal deaths.
